# American Medical College Association

**Published:** 1877-06

**Authors:** 


American Medical College Association.—This institution was permanently organized at the meeting in Chicago, on June 2d.

Prof, Biddle, of Jefferson Medical College, was made President; Prof. N. S. Davis of Chicago, Vice-President; and Prof. Connor of Chicago, Secretary and Treasurer.

Constitution, by-laws, and articles of confederation were adopted. The by-laws provide that colleges may have membership in the Association or merely affiliation, on certain terms and conditions.

About twenty-five colleges were represented at the meeting, and most of their delegates signed the constitution and by-laws and articles of confederation.

It is believed that but few, if any, of the colleges will fail to affiliate, as the terms are so arranged as to make a fair compromise between colleges entertaining views at variance. Our next issue will contain the constitution, by-laws, and articles of confederation, which our space will not allow in this number of the Journal.

